# LEAFY COTYLEDONs: Connecting different stages of plant development

**DOI:** 10.3389/fpls.2022.916831

**Published:** 2022-08-31

**Authors:** Chen Chen, Xinglin Du

**Affiliations:** College of Plant Science, Jilin University, Changchun, China

**Keywords:** LEC1, LEC2, FUS3, ABI3, phase transition, flowering, epigenetic modification

## Abstract

The life of higher plants progresses successively through embryonic, juvenile, adult, and reproductive stages. *LEAFY COTYLEDON* (*LEC*) transcription factors, first discovered in *Arabidopsis thaliana* several decades ago, play a key role in regulating plant embryonic development, seed maturation, and subsequent growth. Existing studies have demonstrated that *LECs* together with other transcription factors form a huge and complex regulatory network to regulate many aspects of plant growth and development and respond to environmental stresses. Here, we focus on the role that has received little attention about the *LECs* linking different developmental stages and generational cycles in plants. We summarize the current fragmented research progress on the *LECs* role and molecular mechanism in connecting embryonic and vegetative growth periods and the reproductive stage. Furthermore, the possibility of *LECs* controlling the maintenance and transition of plant growth stages through epigenetic modifications is discussed.

## Introduction

The plant lifecycle goes through embryonic, juvenile, adult, and reproductive stages. Transitions between different developmental stages of plants ensure that plants can complete the lifecycle and maintain normal growth and development and adaptation to environmental stresses.

The embryonic stage, which begins with the formation of a single-celled zygote after the fertilization of an egg by a male gamete, can be divided into morphogenesis, maturation, and dehydration (Cridge et al., 2016). Forward genetics methods have identified a series of the master genes *LAFL* that regulate embryonic development, including *LEAFY COTYLEDON1* (*LEC1*) and *LEC1-LIKE* (*L1L*) of the *NF-YB* gene family, and *ABSCISIC ACID INSENSITIVE3* (*ABI3*), *FUSCA3* (*FUS3*), and *LEC2* (*LEAFY COTYLEDON2*) of the *B3-AFL* gene family (Giraudat et al., 1992). *LEAFY COTYLEDONs* (*LEC1, LEC2,* and *FUSCA3*) are the key genes in plant embryonic development. During embryonic morphogenesis, the *LEAFY COTYLEDONs* (*LECs*) determine the fate of stalk cells and control cotyledon characteristics. In the later stage of embryonic maturation, *ABI3* promotes the accumulation of storage substances and increases the dehydration resistance of embryos (Giraudat et al., 1992; Meinke, 1992; Meinke et al., 1994; West et al., 1994). Similar to *LEC1*, *L1L* is expressed primarily during seed development. However, suppression of *L1L* gene expression induced defects in embryo development that differed from those of *lec1* mutants, suggesting that *LEC1* and *L1L* play unique roles in embryogenesis (Kwong et al., 2003). Furthermore, *LEC1* and *L1L* genes act as key regulators to coordinate the expression of fatty acid biosynthetic genes (Mu et al., 2008). Loss of function of each of these genes produced distinct phenotypes, with enhanced phenotypes in double mutants, suggesting that these genes regulate embryonic development through distinct but redundant pathways. Subsequent studies found that the *LEC1* encodes a transcriptional activator that is mainly expressed in seeds and encodes a HAP3 subunit belonging to the HAP (NF-Y or CBF) family of transcription factors, which binds the CCAAT-box. The HAP complex includes three distinct subunits: HAP2/NF-YA/CBF-B, HAP3/NF-YB/CBF-A, and HAP5/NF-YC/CBF-C, and HAP3 plays an important role in plant embryonic development, chlorophyll biosynthesis, flowering regulation, etc. (Lotan et al., 1998), whereas *LEC2*, *FUS3*, and *ABI3* all belong to the AFL subfamily of the LAV family in the B3 transcription factor superfamily; hence, they are called AFL-B3 regulators (Luerssen et al., 1998; Stone et al., 2001). *LEC2*, *FUS3*, and *ABI3* can activate downstream target gene expression by binding the conserved region of B3 to the RY/Sph conserved sequence (CATGCAT) cis-regulator. Numerous studies have shown that these genes are involved in embryonic development and need to be inhibited in the vegetative phase after seed germination to ensure that the plant can enter the vegetative phase smoothly (Bentsink and Koornneef, 2008). Several repressors, including chromatin remodeling proteins (Henderson et al., 2004; Tang et al., 2008), and DNA-binding transcription factors (Moon et al., 2003) are involved in suppressing the expression of embryonic development genes in vegetative organs. Therefore, plant seeds can smoothly enter the vegetative growth stage after germination. The traditional view is that the embryonic stage is the period when the stem and root meristems are initially formed (Tsukagoshi et al., 2007), whereas the juvenile stage in vegetative growth is when the shoot apical meristem begins to form stems, true leaves, and lateral buds (Poethig, 1990). However, for some subterranean sprouting plants, such as beans and maize, a certain number of leaves are already present during embryogenesis, indicating that the juvenile stage begins during embryonic development. The above results show that there is a close relationship between plant embryonic development and vegetative growth. However, research in this area is relatively fragmented and there is a lack of sufficient research on how *LECs* regulates vegetative growth. Hence, the underlying molecular mechanisms and the significance of low-level expression of *LEAFY COTYLEDONs* during vegetative periods remain unclear and are both discussed thoroughly in this study. For the first time, we have systematically discussed the key role of *LEAFY COTYLEDONs* in regulating embryonic and vegetative stages, in order to provide new ideas for researchers to further study the relationship between embryonic and vegetative stages.

The vegetative growth period includes juvenile and adult stages. The transition of plants from juvenile to adult is called the vegetative phase change (VPC; Huang et al., 2015a), whereas flowering represents the transition from plant adulthood to the reproductive stage. And there are many studies on the relationship between vegetative growth and reproductive growth.

Many perennial overwintering plants undergo vernalization to flower (Xu and Chong, 2018), cycling through embryonic, juvenile, adult, and reproductive stages. In *Arabidopsis thaliana*, repression of flowering *via* chromatin modification (lysine trimethylation at position 27 of histone H3–H3K27me3; Bouché et al., 2017; Xu and Chong, 2018). The vernalization-induced *FLC* silencing is stable in the current generation, but the next generation needs to reactivate the expression of *FLC* to ensure that the plants are vernalized through cold winters in each generation, thereby preventing the plants from blooming before or during the winter (He and Li, 2018). It has been found that the seed-specific “pioneer” transcription factor *LEC1* initiates this reset process after fertilization and cooperates with *LEC2* and *FUS3* to form a “vernalization memory” embryo reset pathway, thereby activating the expression of *FLC* (He and Li, 2018) so that the plant can smoothly re-enter the embryonic stage from the reproductive stage for the generation cycle.

Here, we focus on *LEAFY COTYLEDONs* (*LECs*) and elucidate the molecular and epigenetic mechanisms of embryonic developmental regulators that play a role in linking different developmental stages to regulate the plant life cycle. In addition, previous studies were mostly limited to the function of *LEAFY COTYLEDONs* at a specific developmental stage. Hence, little attention has been paid to the role of *LEAFY COTYLEDONs* in plant phase transition and life cycle, and there is a lack of systematic and in-depth research on its molecular mechanism. We further examine the molecular mechanisms by which *LECs* regulate plant phase transitions and propose a possible model of the complete life cycle.

## Plant phase transition and distinguishable boundaries

The plant cycle goes through the embryonic stage, the vegetative growth period, and the reproductive growth period (also called the flowering period). Dicotyledonous embryogenesis starts from the fertilized egg (zygote), which is divided into several morphologically distinct stages: proembryo, heart-shaped embryo, spherical embryo, torpedo-shaped embryo, and horseshoe-shaped embryo (De Smet et al., 2010). After embryonic morphogenesis, seeds enter a period of desiccation and then dormancy. Previous studies have found that seed development strictly follows a specific order, which is regulated by a series of embryonic development regulators *LEAFY COTYLEDONs* (such as *LEC1*, *LEC2*, *FUSCA3*, and *ABI3*) that are activated in sequence (Raz et al., 2001).

After morphogenesis, the embryo differentiation and development need to be arrested in time to ensure that the seeds enter the desiccation phase smoothly and acquire desiccation tolerance. The embryo growth arrest is regulated by the *FUS3/LEC* type genes to enable the seed to enter the maturity stage, as mutations in these genes cause a continuation of growth of immature embryos (Raz et al., 2001). Furthermore, *LEC1*, *LEC2*, *FUS3,* and ABI3 can cooperate with each other to activate target genes (Santos-Mendoza et al., 2008) and thus participate in the signaling pathway of ABA-mediated seed maturation regulated by ABI3 and ABI5 (Suzuki and McCarty, 2008).

After embryonic development, the radicle and plumule break through the seed coat, and the cotyledons unfold to allow photosynthesis, signifying that the plant is entering the vegetative growth phase. Juvenile and adult stages have easily distinguishable morphological features in the model plant Arabidopsis. Juvenile leaves are small and round, with smooth and non-notched edges, and epidermal hairs on the adaxial surface (Tanaka et al., 2008). Previous studies have found that *miR156* is a master control factor in the transition from juvenile to adult stage and is evolutionarily conserved (Wang et al., 2009). The *miR156* controls the leaf shape and epidermal hair traits of plants by regulating the *SQUAMOSA promoter-binding protein-like9* (*SPL9*) gene family (Wu et al., 2009). When the vegetative growth enters the final stage, the plant reaches mature/adult state, whereby the expression of a series of genes changes under the stimulation of seasonal and temperature signals. For example, the inhibition of flowering by *FLC* is relieved (Searle et al., 2006) and the floral meristem-determining gene *LFY* is upregulated (Blázquez et al., 1997), simultaneously initiating flower buds to begin differentiation, which marks the beginning of the reproductive growth phase (Blázquez et al., 1997). In addition, a series of hormonal regulation and epigenetic modifications are also involved in different stages of this transition from vegetative to reproductive growth.

## LEAFY COTYLEDONs are inhibited to ensure that the plant smoothly enters the vegetative growth stage

After entering the vegetative growth phase, the expression of the whole plant genome changes. Some genes that are highly expressed in the embryonic stage, such as *LAFL*, need to be suppressed to ensure that the plant can enter the vegetative phase smoothly (Jia et al., 2014). Several epigenetic modifications and transcription factors play an important role (Jia et al., 2014).

Chromatin remodeling complexes *POLYCOMB REPRESSIVE COMPLEXES* (*PRC*) *1* and *2* are required for establishing and maintaining stable epigenetic repression in response to developmental or environmental signals (Mozgova and Hennig, 2015). Briefly, *PRC2* has a histone 3, lysine 27 trimethylation (H3K27me3) activity, and *PRC1* can recognize H3K27me3 and lead to chromatin compaction through histone H2A lysine ubiquitination (H2Aub; Bouyer et al., 2011). The LAFL genes are repressed by the Polycomb machinery *via* its histone-modifying activities, including the histone H3 K27 trimethyltransferase activity of the PRC2 and the histone H2A E3 monoubiquitin ligase activity of the PRC1 during seed germination (Makarevich et al., 2006; Bratzel et al., 2010; Yang et al., 2013; Schneider et al., 2016). The *PCR2* is the main factor involved in cell identity to maintain stable repression of embryogenesis program genes (Salaün et al., 2021).

*VAL1* and *2* (*VIVIPAROUS/ABI3LIKE*) are transcriptional regulators containing DNA-and chromatin-binding domains, and the expression of VAL1 and 2 increased in this process as embryo-repressed genes (Yuan et al., 2021). Specifically, VAL proteins recruit histone deacetylase 19 (HDA19) and *PRC1* to the chromatin regions, which contain genes involved in the regulation of development and dormancy. Thereby, HDA19 removes histone acetylation marks, whereas *PRC1* incorporates monoubiquitinated histone H2A (H2Aub) marks to initiate initial repression of the target gene (Makarevich et al., 2006). Thus, the VAL proteins appear to cause the initial repression of the seed-development and germination-related genes. Later on, this repression is maintained by PRC2-mediated trimethylation at H3K27 (Makarevich et al., 2006). It is critical to note that *VAL1* was shown to interact with HDA19 and to repress LAFL gene expression during germination (Zhou et al., 2013).

The *PICKLE* genes (*PKL* and *PICKLE RELATED 1* and *2*—*PKR1* and *PKR2*) are also upregulated in the process, and they are members of the CHD3 family of chromatin ATPase remodelers (Han et al., 2015). It was found that PKL acts throughout the seedling, repressing the expression of embryonic traits, and is required for GA-dependent responses in shoots (Henderson et al., 2004; Smolikova et al., 2021). Furthermore, in the *pkl* and *val1* mutants, the chromatin-based repression of *LAFL* is less important and, consequently, both *PKL* and *VAL* have a repressive effect on the expression of *LAFL* genes during zygotic embryo development (Hurtado et al., 2006; Bouyer et al., 2011; Han et al., 2015; Shen et al., 2015).

miRNAs also play an important role in the process of transitioning from embryonic to the vegetative growth phase. The *miR156-SPLs* control the transition from juvenile to adult stage and are evolutionary conserved (Morea et al., 2016). The expression of *miR156* decreased gradually with age, whereas the expression pattern of *SPL* was negatively correlated with age (Kim, 2020). The miRNAs were shown to target *LEC1*, *LEC2,* and *FUS3* directly or indirectly and to suppress their activities in the embryo during embryo development, until they are required (Wu and Poethig, 2006; Willmann et al., 2011). Interestingly, LAFL genes such as FUSCA3 can target miR156A and miR156C to activate miR156, thus delaying the VPC (Lumba et al., 2012; Vashisht and Nodine, 2014). In summary, the expression of miRNA inhibited *LAFL* to ensure the smooth entry of plants into the vegetative growth phase, and for the transition of the juvenile to adult phase.

## Function of LEAFY COTYLEDONs during vegetative growth

*LECs* have been extensively studied as regulators of embryonic development and seed maturation. However, little is known about how they regulate postembryonic development and vegetative growth.

LEC1 encodes the B subunit of NF-Y that consists of NF-YA, NF-YB, and NF-YC, and often functions as a pioneer transcription factor (Lotan et al., 1998; Wang et al., 2011, 2012; Nardini et al., 2013; Wang and Perry, 2013) and is first activated after pollination and is expressed in both embryo and endosperm. Song et al. found that an embryonic development arrest, caused by loss of *LEC1* function in the embryo, can be rescued by normally expressed *LEC1* in the endosperm (Fleming et al., 2013). The loss of *LEC1* function in the endosperm leads directly to the embryonic development arrest and cannot be rescued by *LEC1* in the embryo (Fleming et al., 2013). Therefore, *LEAFY COTYLEDONs* also play a crucial role in tissues other than embryos in plant growth and development. Hou et al. found that hypocotyl elongation was inhibited in the *lec1* mutants relative to wild type in Arabidopsis (Oldfield et al., 2014). *LEC1* and *PHYTOCHROME-INTERACTING FACTOR4* (*PIF4*) have a direct interdependent relationship, and can jointly bind to the promoter G-box elements of the hypocotyl elongation-related genes such as *INDOLE-3-ACETIC ACID INDUCIBLE 19* (*IAA19*) and *CYTOKININ INDUCED ROOT CURLING 2* (*YUC8*), and coordinately regulate hypocotyl elongation (Oldfield et al., 2014). In addition, Hou et al. also revealed that *LEC1* inhibited the development of cotyledon trichomes by regulating the expression of the trichome development-related genes such as *GLABROUS1* (*GL1*), *GL2*, and *GL3* and recruiting the transcription factor *TRICHOMELESS2* (*TCL2*; Huang et al., 2015a).

AFL-B3 regulators (*FUSCA3*, *LEC2*, and *ABI3*) have also been shown to be associated with root development. In culture medium free of any exogenous hormone supplementation, while adventitious root emergence and growth was prominently observed in wild-type cells, no such features were observed in *abi3-6* cells (Sherwood et al., 2014). Expression of auxin-responsive *AUX1* and *GH3* genes was significantly reduced in *abi3-6* cells, indicating that auxin levels or distribution may be altered in absence of *ABI3* (Sherwood et al., 2014). In addition, the function of auxin in promoting lateral root formation may be related to *FUSCA3* and *LEC2*, and these two homologous B3 transcription factors have been shown to interact to bind to the auxin biosynthesis gene *YUCCA4* (*YUC4*) and synergistically activate its transcription during lateral root formation (Song et al., 2021). Furthermore, *FUSCA3* expression is activated by *LEC2* in lateral root initiation. The observations indicate that the FUS3-LEC2 complex functions as a key regulator in auxin-regulated lateral root formation (Song et al., 2021).

These findings suggest that *LEAFY COTYLEDONs* play an important role in regulating embryonic development and vegetative growth.

## LEAFY COTYLEDONs regulate the vegetable phase change

During the transition from juvenile to adult stage, plants are accompanied by multiple physiological and biochemical metabolic processes, such as biomass, reproductive capacity, resistance to pests and diseases, stress resistance, and plant architecture (Chuck et al., 2011; Huang et al., 2015b; Tang et al., 2017; Sengupta and Nag Chaudhuri, 2020). Previous studies have shown that *LEAFY COTYLEDONs* play an important role in regulating plant stage transitions.

The *FUSCA3* is a transcription factor belonging to the B3 family and plays an important role in the determination of embryonic leaf identity and the timing of development (Luerssen et al., 1998). Whole-genome microarray analysis found that the expression of the genes related to ethylene signaling pathway increased in *fus3* mutants, including *ACS6*, *ERF1*, *EDF4*, *EDF2*, etc. (Lumba et al., 2012). The loss of *FUSCA3* function resulted in advanced vegetative phase change and cotyledon replacement by leaves (Vashisht and Nodine, 2014). It has been proven that *FUSCA3* may partially depend on the ethylene signaling pathway to regulate the vegetative stage transition. In seeds, *FUSCA3* binds to and represses *AtGA3ox2*, reducing levels of bioactive GA in tissues where it is expressed, thus repressing germination (Wang et al., 2012). *Dwarf8*, a DELLA protein that, when mutated, results in shortening of a delay in VPC, is downregulated along with another DELLA (Wang et al., 2012).

In addition, *FUSCA3* can be phosphorylated by SNF-related protein kinases (SnRKs) in Arabidopsis, and exogenous application of JA delays VPC and rejuvenizes leaf five in both epidermal characteristics and gene expression patterns (Wang et al., 2012). Using Genome-Wide Identification of *in Vivo* Binding Sites for *FUS3*, it was found that *FUSCA3* can directly bind to the promoters of *miR156A* and *miR156C* (two major precursors of *miR156*; Lumba et al., 2012). The *miR156-SPL* is the master pathway regulating vegetative stage transition. The *miR156* inhibits *SPL3*, *SPL4*, *SPL5*, *SPL9*, *SPL10*, and *SPL13*, and targets *miR172* (a microRNA that represses the AP2 gene); this pathway mechanism is evolutionary conserved (Schneider et al., 2016). To sum up, *FUSCA3* cooperates with ABA/GA during the embryonic stage to regulate the transition from embryonic to the vegetative stage, whereas the function of *FUSCA3* in the vegetative stage regulates the transition of the vegetative to reproductive stage by coordinating the regulation of the ethylene signaling pathway and balancing the two hormones JA and GA. Therefore, *FUS3* may regulate VPC through two pathways (*via* hormones and *via miR156-SPL*). However, it remains unclear whether these two pathways cross and what their molecular mechanisms are.

In Arabidopsis, *Polycomb repressive group complex 2* (*PRC2*) inhibits gene expression by catalyzing the trimethylation of histone H3 at lysine 27 (H3K27me3; Wang et al., 2012). The major seed regulatory genes, including *LEC2*, *FUSCA3*, and *ABI3*, were found to be enriched in the *CURLY LEAF* (*CLF*) and *SWINGER* (*SWN*) binding regions through ChIP seq analyses to profile the genome-wide distributions of *CLF* and *SWN* in Arabidopsis seedlings (Cui et al., 2014).*CLF* and *SWN* are the two major H3K27 methyltransferases and are the core components of *PRC2*, targeting transcripts of MADS-box transcription factors and *SPLs*, and those of *miR156A/C* and *MIR172*, as well as playing roles in VPC (Cui et al., 2014). Furthermore, in contrast to wild type with abaxial trichomes on leaf 7, the *swn-3* mutant exhibited the phenotypes with significantly delayed vegetative phase change and phenotypes with later abaxial trichomes on leaf 11 and rounder leaves; by contrast, *clf29* had similar trichome production and leaf shape to wild type (Mao et al., 2017). Even though *clf-29* did not exhibit a vegetative phase change phenotype, we cannot rule out the possibility that *CLF* acts redundantly with *SWN* in influencing vegetative phase change (Mao et al., 2017). Hence, we can hypothesize that *LEAFY COTYLEDONs recruit SWN or CLF in miR156 loci to influence VPC.* It is worth noting that miR156 has an age-dependent expression pattern because it is highly expressed in young plants and gradually decreases with age. Therefore, the miR156 must have undergone a reset process during the generational change. The latest research shows that LEC2 is involved in regulating the transgenerational reset process of miR156A/C in the late embryo to ensure the smooth lifecycle of plants (Osadchuk et al., 2021). However, the research on the regulation of VPC by *LECs* is still relatively small, and more attention needs to be devoted to this area.

## LEAFY COTYLEDONs participate in the regulation of plant flowering *via* the vernalization pathway

Many over-wintering plants in temperate climates acquire the competence to flower in spring after experiencing prolonged cold exposure through a process known as vernalization (Bouché et al., 2017; Xu and Chong, 2018). The vernalization pathways shut down the expression of a potent floral repressor and this vernalization-mediated repression or “vernalized state” is maintained in cell divisions during subsequent growth and development when the temperature rises in spring, named epigenetic “memory of winter cold,” enabling plants to flower in spring (Xu and Chong, 2018; Shu et al., 2019). However, the “memory of winter cold” must be reset in the next generation to ensure that each generation or each growth cycle experiences winter cold before flowering and thus flowers at the right season to maximize reproductive success (He and Li, 2018; Shu et al., 2019). In *Arabidopsis thaliana*, the vernalization pathway blocks the expression of the potent floral repressor *FLC* to enable spring flowering (Xu and Chong, 2018). Recent studies reveal that shortly after fertilization *LEC1* functions as a pioneer transcription factor to initiate *FLC* resetting/re-activation, and that *LEC2* and *FUS3* subsequently function together with *LEC1* to fully re-activate *FLC* expression in early embryogenesis by recruiting the plant-specific scaffold protein *FRI*, which physically associates with the H3K36 methyltransferase *Embryonal Fyn-associated substrate* (*EFS*) and other active chromatin modifiers (Xu et al., 2016; Gao et al., 2022). Thus, *LEC1*, *LEC2*, and *FUSCA3* govern the reprogramming process of vernalized memory. Interestingly, *VAL1/2* belongs to the B3 family of transcription factors as *FUSCA3* and *LEC2*, and both can bind to the cold memory element CME of *FLC*. Therefore, after the plants entered the vegetative phase, the expression of these embryonic developmental genes decreased, and *VAL1/2* competed to bind to the *FLC* promoter motif to shut down *FLC* expression, allowing plants to flower in warm spring (Xu et al., 2016). Therefore, these three *LEC* genes reset the parental “memory of winter cold” during early embryogenesis in Arabidopsis and indirectly involved in repressing *FLC* to allow flowering, and this is of great significance for plants to successfully complete the generational alternation (Andrés and Coupland, 2012; Niu and He, 2019).

## LEAFY COTYLEDONs regulate phase transitions and complete the lifecycle through epigenetic modifications

Epigenetic modifications are involved in the phase transition and lifecycle of plants, and *LECs* play an important role in them.

**Figure 1:**
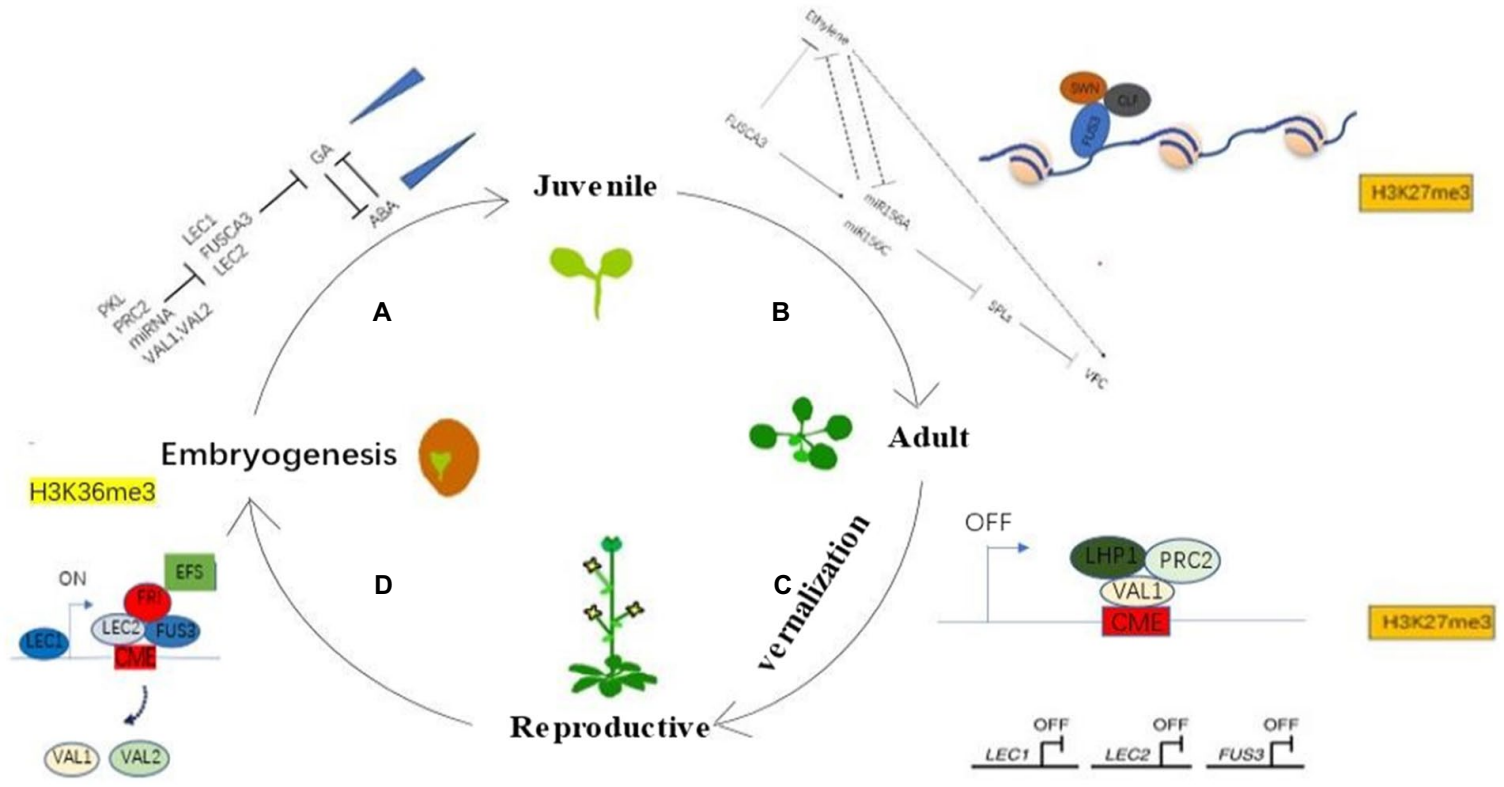
*LEAFY COTYLEDONs* connect different growth stages of plants and complete the generation cycle through epigenetic modification and mediating phytohormonal signaling. **(A)** *LEAFY COTYLEDONs* regulate the transition from embryogenesis to juvenile stage. *PKL*, *PRC2*, miRNA, and *VAL1/VAL2* inhibit the expression of *LEAFY COTYLEDONs* to inhibit the GA signaling pathway. GA is antagonistic to ABA, and the expression of GA increases continuously, whereas ABA shows the opposite expression pattern during this stage. **(B)** LEAFY COTYLEDONs regulate the transition from juvenile to adult stage. There are potentially two pathways of how *LEAFY COTYLEDONs* regulate the transition from juvenile to adult stage. One is that *FUS3* promotes VPC by recruiting chromatin repressors such as *SWN* and *CLF* for chromatin modification of *miR156* to inhibit the expression of *miR156* and then promote the expression of downstream *SPLs*. Another is that *FUS3* regulates VPC by participating in the ethylene signaling pathway, and there may be a cross-talk between these two pathways. **(C)** Perennials experience vernalization from juvenile to adulthood. *VAL1/2* competes with *LEAFY COTYLEDONs* for binding to the *FLC* cold memory element CME, and *VAL1/2* shuts down *FLC* expression by recruiting *PRC2* and *LHP1* for chromatin modification of *FLC*, as *LEAFY COTYLEDONs* shut down expression during this period. **(D)** Plants enter embryonic stage after fertilization. The *LEAFY COTYLEDONs* are expressed sequentially, and the plant enters a new life cycle by recruiting active chromatin modifiers such as *FRI* and *EFS* to activate the expression of *FLC* and a series of other genes such as *miR156*. *VAL1/2* are suppressed during this period.

*BRAHMA*(*BRM*) is a *SWI2/SNF2* chromatin remodeling ATPase, and *BRM* antagonizes the PcG protein complexed with *SWN* protein influencing the histone methylation status of the *MIR156A* promoter region (Smolikova et al., 2021). In addition, the *brm* mutants accelerate VPC and flowering in Arabidopsis. Furthermore, *BRM* opposes *PICKLE* at the target loci, whereby *BRM* maintains active expression and *PKL* balances active and repressive states of these targets and promotes H3K27me3, leading up to phase change as *SWI/SNF* chromatin remodelers (Hurtado et al., 2006). In Arabidopsis, the *pkl* mutants delay VPC and flowering, and *PKL* cooperatively interacts with *SWN* or *CLF* to regulate target genes. In addition, the *LAFL* network (*LEC1*, *LEC2*, *ABI3*, and *FUSCA3*), as a negative regulator of seed germination, needs to be suppressed before seedling development. This repressive signal is mediated by *PRC1* and *PRC2*, as well as *PKL* and *PICKLERELATED2* (*PKR2*) proteins (Tao et al., 2019). However, chromodomain, helicase, and DNA-binding CHD1 protein (CHR5) are involved in establishing the active chromatin state of seed maturation genes. *CHR5* is expressed during late embryogenesis, antagonizing *PKL* in embryo gene expression and seed storage protein accumulation (Tao et al., 2017). In addition, *CHR5* was shown to be associated with the promoters of *ABI*3 and *FUS3* and to be required for reducing nucleosome occupancy near the transcriptional start site (Shen et al., 2015). In view of the above, *LEAFY COTYLEDONs* have distinct spatiotemporal expression patterns and create a complex dynamic regulatory network with other transcription factors and epigenetic modifiers and consequently play a key role in connecting different developmental stages of plants. Based on the above, we propose a possible model (Figure 1) of the molecular mechanism of *LEAFY COTYLEDONs* involved in regulating plant phase transition from the macro perspective of plant generation cycle.

## Conclusion

The development of higher plants starts from the zygote, and plants already begin to prepare for subsequent vegetative growth, flowering, and reproduction during embryonic development. However, there are many studies on the relationship between vegetative growth and reproductive growth, but few studies on how the embryonic stage affects vegetative growth and reproductive growth. In fact, epigenetic modification in the embryonic stage allows the plant to remember this modification and pass it on to the postembryonic vegetative and reproductive growth stages. We think that *LEAFY COTYLEDONs* may play a key role in linking different reproductive stages of plants through the establishment of active chromatin during the embryonic stage and the recruitment of chromatin modifiers. The Polycomb repressive group complex (PcG) proteins *EMF1*, *CLF*, *LHP*, and *SWN* are involved in these processes. *FUSCA3* has been shown to perform different functions in embryonic and vegetative stages, synergize with JA, GA, and ABA, and participate in the epigenetic modification process to regulate the stage transition in plants. *LEAFY COTYLEDONs* form a complex network with histone epigenetic modifiers, miRNAs, and transcription factors specifically expressed at each growth stage, and they also mediate phytohormone signals. However, previous studies were mostly limited to the function of *LEAFY COTYLEDONs* at a specific developmental stage, with little attention paid to the role of *LEAFY COTYLEDONs* in plant phase transition and life cycle. However, the progression of the life cycle is critical for perennials. During the cycle of each generation, the reactivation of many genes is required to start a new round of life activities. For example, the flowering suppressor gene *FLC* is reset by *LEAFY COTYLEDONs* during embryonic stage, and the age master gene *miR156A/C* is reset by *LEAFY COTYLEDONs* during embryonic stage. A growing number of studies have demonstrated the critical role of *LEC* genes in altering plant generations, but these studies are only in their infancy. We hope this paper will provide new research ideas for researchers to study various physiological activities associated with the alteration of plant generations. *LEAFY COTYLEDONs* are involved in many physiological and biochemical processes in plants, especially in epigenetic modifications, which we believe play a crucial role in tandem with different developmental stages in plants. We propose a model for the role of *LEAFY COTYLEDONs* in linking different growth stages and completing the generational cycle in this study (Figure 1). Further exploration of *LEAFY COTYLEDONs*’ intrinsic molecular mechanism has far-reaching significance for understanding the principle of the plant lifecycle and applying it to agricultural production.

## Author contributions

CC and XD contributed to the conception and design of the study. CC wrote the first draft of the manuscript. XD make critical revisions to the work. All authors contributed to the article and approved the submitted version.

## Conflict of interest

The authors declare that the research was conducted in the absence of any commercial or financial relationships that could be construed as a potential conflict of interest.

## Publisher’s note

All claims expressed in this article are solely those of the authors and do not necessarily represent those of their affiliated organizations, or those of the publisher, the editors and the reviewers. Any product that may be evaluated in this article, or claim that may be made by its manufacturer, is not guaranteed or endorsed by the publisher.
